# Ros-mediated mitochondrial oxidative stress is involved in the ameliorating effect of ginsenoside GSLS on chlorpyrifos-induced hepatotoxicity in mice

**DOI:** 10.18632/aging.204298

**Published:** 2022-09-22

**Authors:** Hongyan Pei, Silu Liu, Jianning Zeng, Jinze Liu, Hong Wu, Weijia Chen, Zhongmei He, Rui Du

**Affiliations:** 1 College of Chinese Medicinal Materials, Jilin Agricultural University, Changchun 130118, China

**Keywords:** CPF, L02 cell line, liver injury

## Abstract

Chlorpyrifos (CPF), as an extensively used organophosphorus pesticide, often remains on food surfaces or contaminates water sources. CPF can cause many toxic effects on human production and life. As an additional product of non-medicinal parts of ginseng, the pharmacological activity of ginseng stem and leaf total saponin (GSLS) has been verified and applied in recent years. This study aimed to evaluate the protective effect of GSLS on CPF-induced liver damage in mice. Experimental results *in vivo* demonstrate that GSLS can reduce the accumulation of oxidation product MDA by relieving CPF-induced liver function indicators in mice and enhancing the antioxidant enzyme SOD and CAT activities of mice. With the decrease in mRNA expression of BAX, NF-KB, and TIMP in liver tissues, the mRNA expression of Nrf-2, HO-1, and XIAP increased. Through anti-inflammatory, antioxidant, anti-inflammatory and other effects, cpf-induced hepatotoxicity can be alleviated by GSLS. *In vitro* experiments have proved that GSLS can show the ability to scavenge DPPH free radicals and hydroxyl radicals. In addition, GSLS can alleviate chlorpyrifos-induced ROS accumulation in L02 cells, alleviating cytokinetic potential reduction. In summary, by fighting oxidative stress, GSLS can alleviate liver damage caused by CPF.

## INTRODUCTION

Liver is one of the most critical organs involved in the metabolic process of the body, regulating metabolism, secretion and storage as well as other basic physiological functions [1]. Meanwhile, the liver plays a key role in the detoxification and excretion of exogenous and endogenous substances [2]. Clinically, liver injury is a common disease, and its causes are associated to biological factors, chemical factors and autoimmune disorders [3]. Liver injury can further lead to major diseases including liver fibrosis, cirrhosis and liver cancer, posing a great threat to human life and health [4]. On this basis, we should develop more drugs that can prevent liver diseases from the perspectives of low toxicity, high safety and strong efficacy.

Pesticides are often applied to kill insects and weed, thus ensuring global food supplies. In addition to being used in agriculture, pesticides are also used in other fields, or for household use [5]. Actually, humans come into contact with pesticides every day in a different way: by professional farmers [6] or based on contaminated food through the food chain [7]. The use of exogenous pesticide insecticides often exhibits a stronger insecticidal effect than previous pesticides. As a result, these pesticides have made interesting progress in crop yield, quality and pest resistance [8, 9]. Pesticides enter organisms through three main routes: inhalation (lungs), ingestion (mouth), and contact (skin) [10]. Organic phosphoric acid pesticides (OP) are widely used in agricultural production, which have adverse effects on human health, and are also classified as toxicological Class I (highly toxic) by the US Environmental Protection Agency [11, 12]. In our daily life, OP poisoning caused by occupational or accidental exposure remains a major global mortality and morbidity problem [13]. Among them, acute OP severe cases also include suicide, homicide and overdose [14]. At present, chlorpyrifos (CPF) (O, O-diethyl-O -(3,5, 6-trichloro-2-pyridyl) phosphate thioester) is one of the most widely used organophosphorus pesticides globally [9]. It has been confirmed that CPF can cause oxidative stress damage, which is related to the accumulation of lipid peroxidation products in multiple organs of the body [15]. CPF also alters the activity of cellular antioxidants including superoxide dismutase (SOD), glutathione peroxidase (GPx) and peroxidase (CAT) [16]. Therefore, it may generate oxidative stress damage of various organ systems [17]. CPF exposure can cause serious damage to the liver and kidneys.

Ginseng, Panax Gingen root Meyer, is one of the most widely used herbs in the world. With a long history and much attention, it is currently considered to be the most effective natural product of Chinese herbal medicine [18]. Ginseng has a variety of pharmacological activities on central nervous system, cardiovascular system and immune system [19].

Ginseng glycosides, or ginsenoside, is considered to be one of the main active ingredients in roots with a variety of pharmacological activities, including anti-inflammatory [20], antioxidant [21] and regulation of immunomodulatory activity [22]. In addition, ginseng and ginsenosides are also effective in improving blood flow and protecting against cardiovascular dysfunction. The aerial parts of ginseng are non-medicinal parts, mainly stems and leaves, which are usually discarded. Over the past years, most studies on ginsenosides have concentrated on the root or rhizome. However, ginsenosides in the stem and leaf of ginseng (GSLS) are rich in ginsenosides and the components are different from those in the root [23]. Therefore, it is expected to have different biological activities and pharmacological effects.

Currently, there are few reports on the prevention of CPF-induced liver injury and toxicity of GSLS with broad research prospects. This study was carried out *in vivo* and *in vitro*. At first, antioxidant experiments were conducted *in vitro* to determine the scavenging ability of GSLS on DPPH free radical and hydroxyl free radical, and then the optimal inhibitory concentration IC50 and GSLS on CPF were determined. Subsequently, the oxidative stress induced by CPF and the preprotective effects of GSLS were investigated by detecting ROS and a series of antioxidant enzyme activities. Flow cytometry, mitochondrial membrane potential, and RT-QPCR were used to detect the mechanism of CPF-induced apoptosis, aiming to evaluate the potential toxicity of CPF-induced oxidative stress in organisms, and whether GSLS can preprotect CPF-induced liver injury, as well as the mechanism during this process. In the *in vivo* experiment, serum biochemical indexes of mice liver were measured, mRNA related factors expression was detected by RT-QPCR, and liver histopathological examination was performed by H&E staining, aiming to evaluate the antioxidant stress ability and liver protection of GSLS as well as to explore its effects on CPF-induced liver toxicity in mice.

## MATERIALS AND METHODS

### *In vitro* experimental materials and methods

#### Material

CPF was purchased from Kaifeng Inspur Chemical Co., LTD, China. CPF prepared CPF reserve solution in dimethyl sulfoxide (DMSO) (Beijing Sorobio Technology Co., LTD, China). During the experiment, the concentration of DMSO was kept below 0.05%. The physiological saline was purchased from Heilongjiang Kelun Pharmaceutical Co., LTD., and the CCK-8 kit, reactive oxygen species kit and PI/ accessory v dye were purchased from That company. All other chemical reagents are of analytical grade and obtained from commercial sources.

Total ginsenosides (GSLS) are provided by Jilin University (Changchun, Jilin, China) and dissolved to final concentration before use. Normal saline was purchased from Heilongjiang Kelun Pharmaceutical Co., LTD, China. ELISA kits for the determination of CAT, MDA and SOD were purchased from Beijing Sorobio Technology Co., LTD, China.

#### Determination of SCAVENGING ability of DPPH free radical

According to the previous research method [1], GSLS solutions with different mass concentrations (0.1, 0.2, 0.4, 0.6, 0.8, 1 mg/mL) were prepared. The positive group received vitamin C solution at a concentration of 50 ug/mL and 25 ug/mL. 2.4 mg DPPH was dissolved in anhydrous ethanol and the volume was kept to 100 mL to obtain the prepared solution of 60 umol/L DPPH. Sample or 70 μL positive drug was added to each well of the 96-well plate. Meanwhile, distilled water was added to the blank well instead of sample solution. 60 umol/L DPPH solution 200 μL was added to each well, and anhydrous ethanol was added to control group instead of DPPH. After the samples were added, the samples were shaken and mixed, and the plates were sealed with glue. After the plates were sealed, the samples were left standing at room temperature for 30 minutes in the dark. The value of A was measured at 517 nm with A microplate reader, and the wells were redrilled for 3 times. The scavenging rate of DPPH free radical was calculated as follows.


$$\text{DPPH clearance rate }\%=\left[1-\left({\text{A}}_{1}-{\text{A}}_{2}\right)/{\text{A}}_{0}\right]\times 100\%$$

A_1_ is the absorbance value of DPPH solution added to the sample to be tested.

A_2_ is the absorbance value of adding anhydrous ethanol solution to the sample to be tested.

A_0_ is the absorbance value of the blank group.

#### Determination of hydroxyl radical scavenging ability

The scavenging ability of GSLS hydroxyl radical was determined. GSLS solutions of different mass concentrations (0.1, 0.2, 0.4, 0.6, 0.8, 1 mg/mL) were prepared respectively. 25 mg heptahydrate was dissolved in water and volume was kept to 10 mL in order to obtain 9 mmoL/L heptahydrate solution. 12.4 mg salicylic acid was dissolved in anhydrous ethanol and reduced to 10 mL to obtain 9 mmoL/L salicylic acid solution. 1 ml 30% H_2_O_2_ solution was added to 100 mL to obtain a solution of 8.8 mmol/L H_2_O_2_. 0.l mL 9 mmol/L heptahydrate ferrous sulfate solution, 0.1 mL 9 mmol/L salicylic acid solution, and 0.2 mL sample solution or positive drug of each concentration were successively added into the 1.5 ml centrifuge tube. In the blank group, 0.2 mL distilled water was used to replace the sample or positive drug, and 1 mL distilled water was added. Finally, 0.1 mL 8.8 mmol/L H_2_O_2_ solution was added, and 0.1 mL distilled water was enhanced to replace H_2_O_2_ in the control group. After the sample was added, the mixture was shaken and taken out in a water bath at 37°C for 30 minutes. 200 μL liquid was added to each well of the 96-well plate. Using an alcohol standard tester, the value of A at 510 nm was determined with 3 times repetition. The calculation formula of hydroxyl radical clearance is presented as follows.


$$\text{Hydroxyl radical scavenging rate }\%=\left[1-\left({\text{A}}_{1}-{\text{A}}_{2}\right)/{\text{A}}_{0}\right]\times 100\%$$

A_1_ is the absorbance value of the sample to be tested by adding H_2_O_2_ solution.

A_2_ is the absorbance value of adding anhydrous ethanol solution to the sample to be tested.

A_0_ is the absorbance value of the blank group.

#### Cell culture and activity determination

L02 human liver shape cells were purchased from the Cell Bank of the Chinese Academy of Sciences (GNR-5, Shanghai, China), and cultured in DMEM complete medium containing 10% FBS in 27°C, 5%CO_2_, and fully saturated humidity incubator. The complete medium was replaced every 48 hours. After the cells grew to cover 80% of the culture flask bottom, they were digested and passed by 0.25% trypsin (containing EDTA).

#### CCK-8 test pesticide IC50 of L02

IC50 is a marker of a substance’s ability to inhibit biological or biochemical processes and represents the concentration of a chemical required for a chemical to inhibit 50% of the activity of its target *in vitro* [24]. The toxicity of CPF on L02 cell activity was evaluated using CCK-8 kit (Shanghai Sheng Biotechnology Co., LTD, China). To obtain different target concentrations, CPF was diluted in DMSO. Then, the density of L02 cells in the medium was adjusted to 1 × 10^6^ cells /mL (100 μL per well) and cultured for 8 hours. After the cells reached the stable state of adherence, CPF with different concentrations (0, 1, 2, 5, 15, 30, 60, 125, 250, 500 μg/ mL) was added to the 96-well plate for 2 hours. Afterwards, the L02 cells were consumed again with 100 μL fresh medium, 10 μL CCK-8 working solution was added to each well, and also cultured for 30 min. The absorbance of each well was measured at 450 nm using a Cytation5 imaging plate reader (Biotek Instrument, USA). Finally, the survival rate of L02 cells was calculated. Using the Logit model, median inhibitory concentration (IC50) and 95% confidence limit were measured.

#### CCK-8 measured the effective concentration of GSLS on CPF-damaged L02 cells

L02 cells with normal activity were inoculated into 96-well culture plates and cultured with 1 × 10^6^ cells/well for 8 h. After cell adhesion, different concentrations of GSLS (0, 30, 60, 120, 240, 480, 600 μg/mL) were added for pre-protection for 12 hours to determine the optimal inhibitory concentration of GSLS. Then, cells were treated with 2 ug/ml CPF in a 96-well plate for 2 hours. Finally, 10 μL CCK-8 reagent was added to each well. After 2 hours, the absorbance of the plate was measured at 450 nm with a microplate reader at 37°C.

#### Detection of reactive oxygen species (ROS)

After cell culture and treatment, we performed the following operations. L02 cells were inoculated into a 6-well culture plate at a density of 1 × 10^5^ cells per well. After 8 hours, the cells were observed to reach a stable adherent state. L02 cells were exposed to CPF for 2 h. Dcfh-da probe was added and incubated at 37°C for 20 min. Subsequently, the cells were washed twice with phosphate buffered saline (PBS, 2.89 g/L Na_2_HPO_4_, 0.2 g/L KH_2_PO_4_, 0.8 g/L NaCl and 0.2 g/L KCl, pH 7.4). The production of ROS was detected by fluorescence microscope (Thermo Fisher Scientific, USA).

#### Mitochondrial membrane potential (Δψm)

Δψm of L02 cells was analyzed using BEYo Time Institute of Biotechnology (JC-1) mitochondrial membrane potential assay kit. JC-1 is applied as a fluorescent probe. In normal healthy cells, Δψm was higher, JC-1 was enhanced, and J-aggregates were formed in the mitochondrial matrix, revealing red fluorescence. Δψm decreased with early apoptosis and JC-1 formed monomer, showing green fluorescence.

L02 cells were inoculated into 6-well plates at a density of 1 × 10^5^/well. After the cells were treated, the cells were suspended in the cell culture medium again and cultured for 20 min with JC-1 staining solution at 37°C. Then, JC-1 dyeing solution was used to clean the plate 3 times. Finally, 500 μL staining solution was added to suspend the cells again.

#### Data analysis

Statistical analysis was performed using GraphPad Prism 9.0 software (GraphPad Software, USA). Two or more groups of experimental data were compared by adopting a one-way anOVA model, and all experimental data were expressed as mean ± standard deviation (standard definition). *P* < 0.05 was considered statistically significant.

### *In vivo* experimental materials and methods

#### Animal and experimental design

Thirty-two 8-week-old male mice were applied in this study. With free access to food and water, they were placed in a laboratory at Jilin Agricultural University at room temperature, with a 12-hour light/dark cycle and 50–70% relative humidity. They could have free access to food and water. The dose of CPF induced liver injury was 10 mg/kg. The selected low dose of the insecticide was based on previous studies in which the 1/20 median lethal dose (LD_50_) of CPF induced biochemical alterations in mice without morbidity [25].

1. Control: 0.9% normal saline was given.
2. CPF group: 10 mg/kg CPF was given orally on day 7 to induce liver injury.
3. CPF+GSLS100: mice were given 100 mg/kg GSLS orally for 7 consecutive days, and GSLS was given 1 hour before oral ADMINISTRATION of CFP (10 mg/kg) for the subsequent 7 days.
4. CPF+GSLS200: mice were given 200 mg/kg GSLS orally for 7 consecutive days, and GSLS was given 1 hour before oral CFP (10 mg/kg) for the subsequent 7 days.

**Figure 1:**
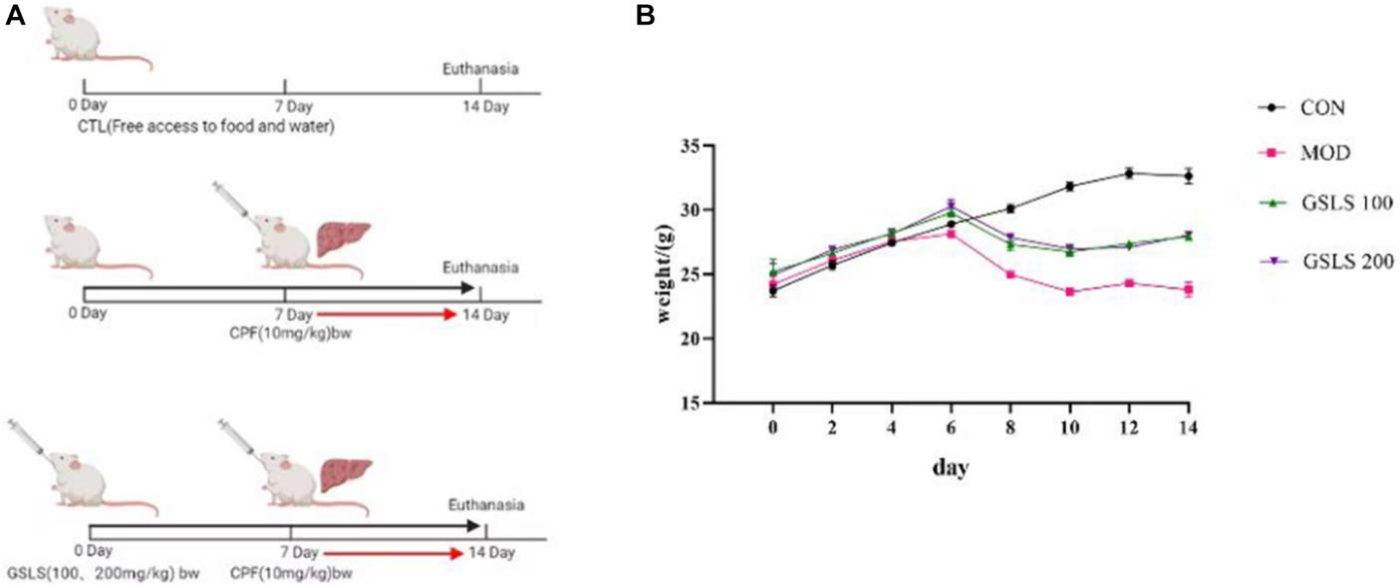
(**A**) Methods of animal administration in each group. (*n* = 8). (**B**) Body weight of mice in each group. (*n* = 8).

The dosage regimen is presented in Figure 1A.

#### General observation

We observed the general situation of each group of animals, including mental, activity, diet and defecation. The mice were weighed every two days during the experiment, and the weight of the mice before oral CPF modeling was applied as the reference value for drug administration.

#### Preparation of liver and kidney homogenates for MDA and antioxidants measurements

At the end of the experiment (14 days), all mice were anesthetized with dimethyl ether and sacrificed by neck dissection. Blood and liver tissue samples were collected. Liver samples were incubated in 0.1 m phosphate buffer (10% w/v) [26] and then centrifuged with the supernatant being frozen at −70°C.The Bio-Rad ELISA reader was used following the manufacturer’s instructions to determine MDA [27], SOD [28] and CAT [29] activity levels in liver homogenate. The experiment was modeled after similar previous studies.

#### Biochemical analysis

Mice liver serum was centrifuged at 4°C, and supernatant was taken after centrifugation at 2500 r/min for 10 min. The levels of ALT, AST and TG in mouse serum were detected by automatic biochemical analyzer. The levels of ALT [30], AST [31] and γGT [32] in serum were determined by colorimetric spectrophotometer.

#### Histopathological examination of liver

Sections of liver tissue were taken from the same part of liver lobe, fixed at 25°C with 4% buffered formalin for 24 h, embedded in paraffin, and cut into 4 μm thick sections. We performed histopathological analysis of liver slices to investigate liver morphology (hemoxysin and eosin staining) and tissue damage/liver fibrosis (Mason’s trichromatic staining). Histopathological changes were observed under light microscope.

#### Quantitative real-time PCR (rt-qPCR) and gene expression

Total RNA was extracted from liver samples and quantified at 260 nm according to device instructions (Bioflux China). CDNA (MyTaq Red Mix, Bioline) was synthesized from 2 μg RNA, and master Mix was amplified by SYBR Green. Table 1 presents the list of primers used for gene amplification. Data were analyzed using the 2δδCt method in CFX96 Touch real-time PCR (Bio-Rad, Inc., USA). Comparative period threshold (CT) values determine changes in gene strength and expression, which can be normalized to β-actin.

**Table 1:** Primers sequence used for quantitative real time PCR in mice in liver.

**Primer sequence**	**Direction**	**Product size (bp)**	**Accession number**	**Gene**
CGCCTGGGTTCAGTGACTCG	Sense	140 bp	NM_010902.4	Nrf2
AGCACTGTGCCCTTGAGCTG	Antisense			
CGCCTCCAGAGTTTCCGCAT	Sense	126 bp	NM_010442.2	HO-1
GACGCTCCATCACCGGACTG	Antisense			
CGCGTGGTTGCCCTCTTCTA	Sense	153 bp	NM_007527	BAX
TTCCCAGCCACCCTGGTCTT	Antisense			
CCCTGACAGGCCACCTGAGA	Sense	153bp	NM_001301641	XIAP
TGAGCATAGTCCGGCCAGTTC	Antisense			
CTTGGTTCCCTGGCGTACTC	Sense	125 bp	NM_007742.4	TIMP
ACCTGATCCGTCCACAAACAG	Antisense			
AAGGATGTCTCCACACCACTG	Sense	139 bp	NM_001177369.1	NFkB
CACTGTCTGCCTCTCTCGTCT	Antisense			
CCAGCCTTCCTTCTTGGGTA	Sense	140 bp	NM_007393.5	β-actin
CAATGCCTGGGTACATGGTG	Antisense			

#### Data analysis

The data of 8 mice in each group were described as mean SEM. SPSS data analysis software was adopted for two-way analysis of variance and Duncan post-descriptive test. *P* < 0.05 was considered statistically significant.

## RESULTS

### *In vitro* experiment results

#### DPPH free radical scavenging effect of GSLS

**Figure 2:**
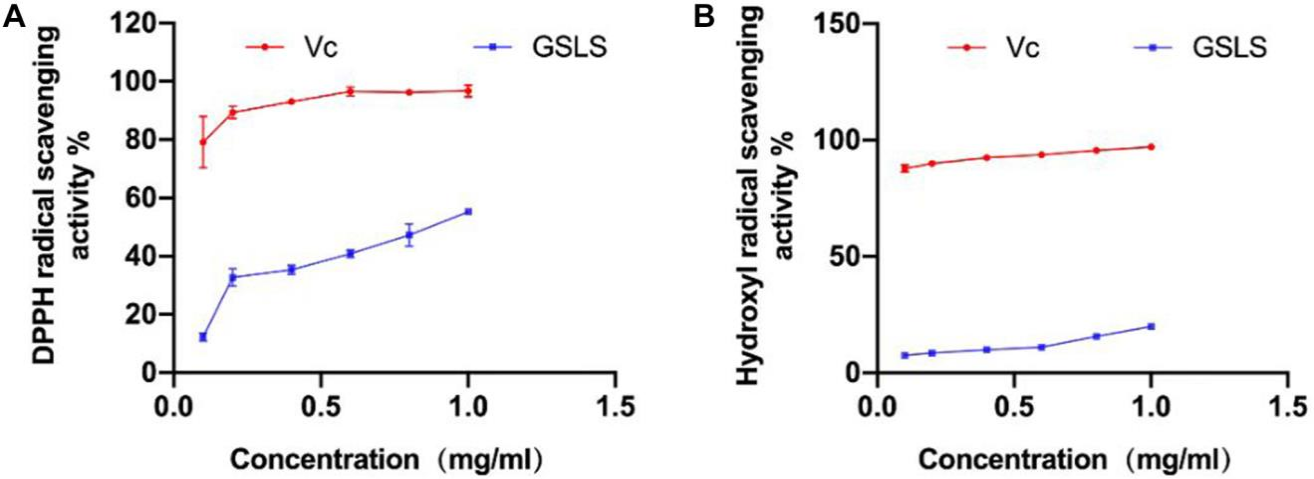
(**A**) Scavenging ability of GSLS on DPPH free radical. (**B**) Scavenging ability of GSLS on hydroxyl free radical.

GSLS has the effect of scavenging DPPH free radical. As shown in Figure 2A, when the concentration of GSLS is between 0.2 mg/ml–0.8 mg/mL, the scavenging ability of DPPH free radical increases significantly, and the scavenging rate increases from 30.09% to 56.01%. Subsequently, the concentration of GSLS increased whereas the scavenging ability enhanced slowly. When the concentration of GSLS increased from 0.8 mg/ml to 1 mg/ml, the scavenging rate increased only approximately 4%, reaching 55.09%. Obviously, GSLS has certain scavenging ability on DPPH free radical.

#### Hydroxyl radical scavenging effect of GSLS

Hydroxyl radical can oxidize salicylic acid and produce colored substances. Based on the principle that the colored products can appear strong absorption peak at 510 nm, the clearance rate of hydroxyl radical is measured and calculated in line with the calculation formula.

As shown in Figure 2B, hydroxyl radical can be cleared by GSLS, and the clearance rate of GSLS on hydroxyl radical increases in a curve. When the concentration of GSLS increases from 0.6 mg/ml to 1 mg/ml, the clearance rate increases from 10.33% to 20.46%. It can be observed that GSLS has certain scavenging ability on hydroxyl radical.

#### Determination of cell viability

**Figure 3:**
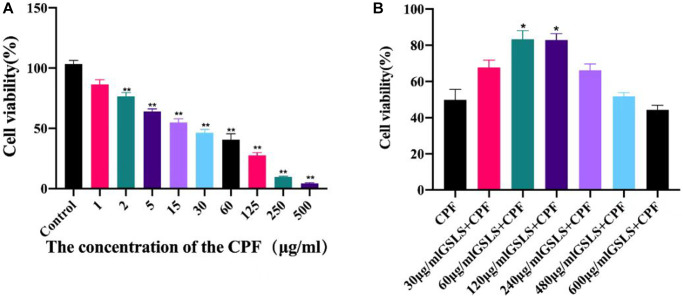
**Cell viability was detected by CCK-8 assay (*n* = 3).** (**A**) The effect of different concentrations of CPF on L02 cells was determined. Usually of The effect of different concentrations of CPF on L02 cells was determined on Cell Cell viability by CCK-8 assay (*n* = 3). (**B**) To determine the optimal protective concentration of GSLS on L02 cells. ^#^*p* < 0.05, ^##^*p* < 0.01 vs. con group; ^*^p < 0.05, ^**^p < 0.01 vs. CPF group.

Exposure to CPF can exert strong toxic effects on organisms. Therefore, the effect of CPF exposure on L02 cell viability was detected by CCK-8. As shown in Figure 3A, different concentrations of CPF inhibited the survival of L02 cells. Thus, CPF exposure decreased cell viability in a concentration-dependent manner. The IC50 of CPF on L02 cells was 15 μg/ mL for 2 hours. After analysis, it was found that when CPF exposure concentration was 2 μg/ mL, the survival rate of L02 cells was significantly different from that of the control group (*P* < 0.05). Therefore, CPF (2 μg/ mL) was selected as the concentration for cell modeling in this experiment.

#### Determination of the optimal inhibitory concentration of GSLS

The effect of CPF on L02 cell viability was detected by CCK-8 method, as presented in Figure 3B. Different concentrations of CPF inhibited the activity of L02 cells. CCK-8 assay was used to explore the alleviating effect of GSLS on CPF-induced L02 cell viability decline. Both 10 μg/mL and 20 μg/mL GSLS significantly decreased cell viability (*P* < 0.05), while other doses showed no significant difference from CPF group. In subsequent experiments, we found that the optimal concentration of GSLS was 10 μg/mL, corresponding to the survival rate of over 90% of L02 cells.

#### GSLS reduced CPF-induced oxidative stress in L02 cells

**Figure 4:**
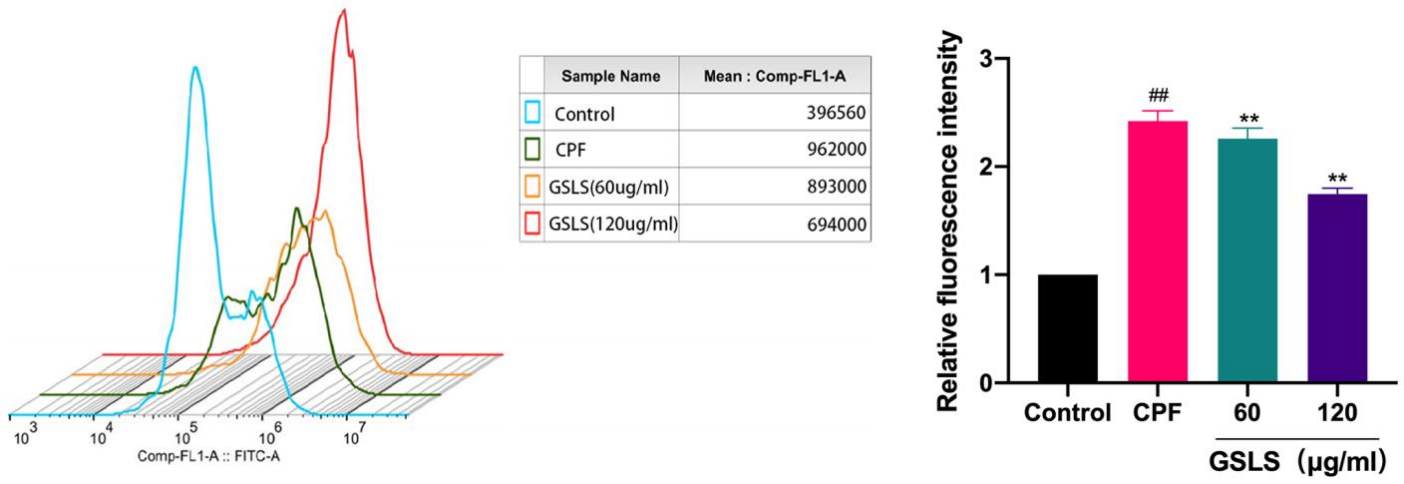
**Flow cytometry analysis of reactive oxygen species (ROS) induced by CPF.** Data are mean ± standard deviation, *n* = 3. ^#^*p* < 0.05, ^##^*p* < 0.01 vs. control group; ^*^p < 0.05, ^**^p < 0.01 vs. CPF group.

ROS are typically produced by mitochondria and are destructive to both the DNA and proteins of pathogens. Studies have revealed that intracellular calcium overload can cause mitochondrial dysfunction, promote the production of intracellular active oxidative cluster ROS, and then lead to cell apoptosis and autophagy [33]. ROS production in L02 cells was detected by fluorescence microscopy (Figure 4). Dcfh-da probe can penetrate cell membrane and hydrolyze into DCFH. Intracellular reactive oxygen species can oxidize non-fluorescent DCFH in order to produce fluorescent DCF.

CPF-treated L02 cells produced more DCF signal than the control group. The experimental data were processed using image J software. Notably, there existed a significant increase in reactive oxygen species (ROS) produced by CPF exposure compared to the control group (*P* < 0.05). Preprotection with GSLS significantly reduced intracellular ROS level, and fluorescence intensity was significantly lower than CPF group (*P* < 0.05). The results suggest that GSLS effectively inhibits cpF-induced intracellular ROS production.

#### GSLS mitigated CPF-induced mitochondrial apoptosis

**Figure 5:**
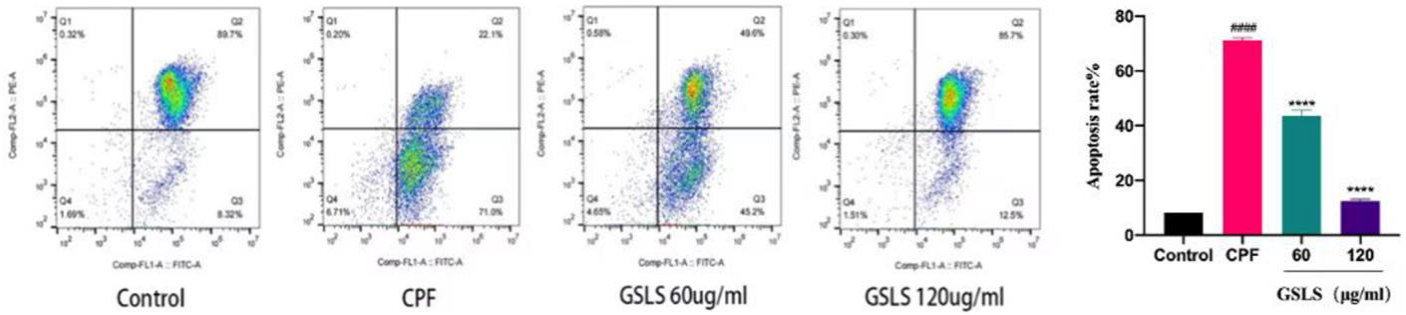
**The detection results of mitochondrial membrane potential (Δψm).** The Δψm staining of each group was JC-1 by flow cytometry. ^#^*p* < 0.05, ^##^*p* < 0.01 vs. control group; ^*^p < 0.05, ^**^p < 0.01 vs. CPF group.

We examined mitochondrial membrane potential to elucidate the mechanism by which CPF exposure induces apoptosis. JC-1 was used as an indicator of Δψm to explore mitochondrial depolarization. As shown in Figure 5, flow cytometry analysis results showed that CPF exposure significantly reduced the red/green fluorescence ratio, indicating that Δψm decreased and mitochondrial depolarization induced apoptosis. Obviously, the red/green fluorescence ratio recovered significantly compared with the GSLS pre-protected group. These results indicated that GSLS saved Δψm, and GSLS could lower mitochondrial dysfunction and effectively inhibit apoptosis induced by CPF exposure.

### *In vivo* experiment results

#### Effects of GSLS on body weight of mice

As presented in Figure 1B, before modeling, the mice lived normally, with shiny color, normal eating and drinking, and a significant weight gain trend. The body weight of mice was measured 1 hour before oral CPF modeling, and there existed no significant difference in body weight among all groups. Mice were preprotected with 100 mg/kg and 200 mg/kg GSLS by intragastric administration. In addition, CPF oral modeling was performed on the seventh day. In addition to the control group, the mice in the rest of the groups were characterized by slow but less eating, at first, hair that was scattered and lost its luster, and weight that dropped significantly. However, when the mice in the GSLS protection group were given the action back, eating normal gradually, weight gradually recovered to steady growth.

#### Effects of GSLS on serum oxidative stress index

**Figure 6:**
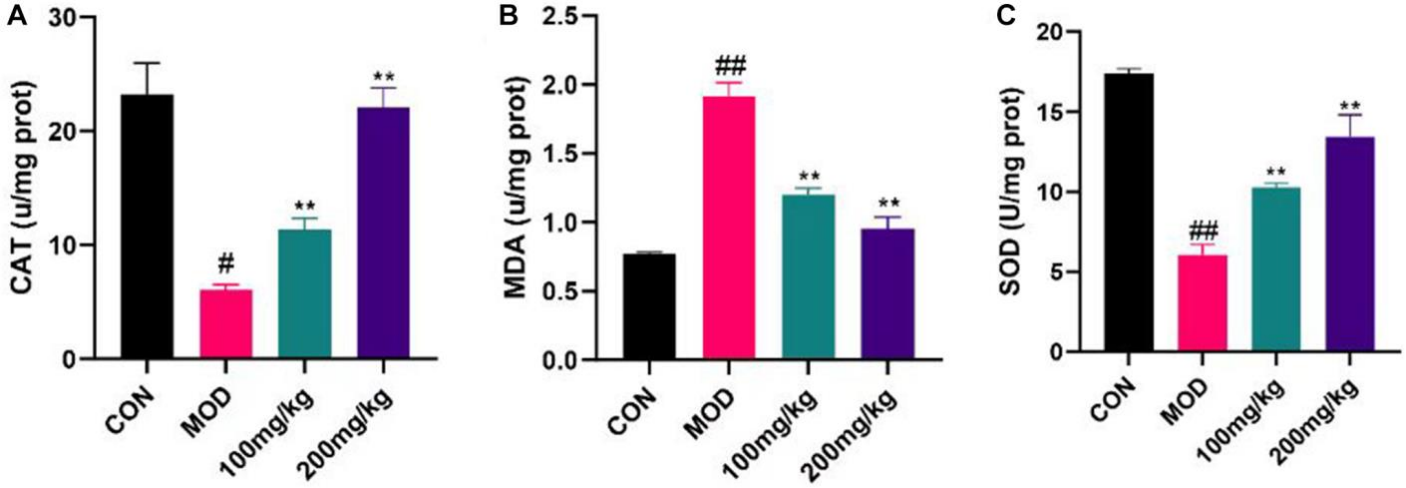
**Regulation of GSLS on oxidative stress in mice.** Liver tissue was crushed into tissue homogenate, and CAT, MDA and SOD levels were detected using kits. (**A**) Content of CAT. (**B**) MDA activity. (**C**) SOD level. ^#^*p* < 0.05, ^##^*p* < 0.01 vs. con group; ^*^p < 0.05, ^**^p < 0.01 vs. MOD group.

As displayed in Figure 6, MDA level of CPF-induced liver injury mice increased significantly, indicating that CPF tissue degenerated and serum SOD and CAT levels decreased. The level of MDA in 100 mg/kg GSLS preprotection group increased whereas SOD and CAT levels recovered. Compared with the control group, the levels of MDA, SOD and CAT in mice pretreated with 200 mg/kg GSLS did not change significantly, proving that GSLS generated a certain alleviating effect on liver injury caused by CPF, and 200 mg/kg GSLS had the best preprotective effect.

#### Serum biological analysis

**Figure 7:**
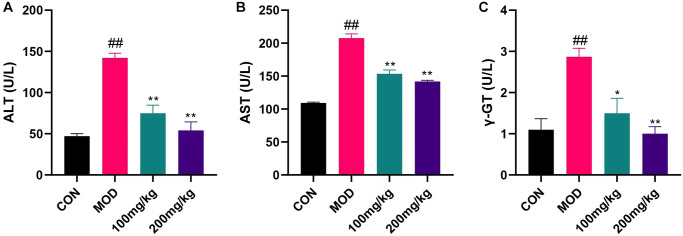
**Changes of liver index induced by CPF in mice.** Serum LEVELS of AST, ALT and γGT were measured with ALT, AST and γGT kits. (**A**) ALT levels in serum of mice. (**B**) Serum AST levels in mice. (**C**) Serum γGT level in mice. ^#^*p* < 0.05, ^##^*p* < 0.01 vs. con group; ^*^p < 0.05, ^**^p < 0.01 vs. mod group.

CPF group showed elevated ALT, AST, γGT and other biomarkers (Figure 7). Before CPF exposure, mice preprotected with GSLS were less influenced by these changes, indicating that the protective effect of 200 mg/kg GSLS was stronger than 100 mg/kg GSLS.

#### Effects of GSLS on quantitative expression of liver genes

**Figure 8:**
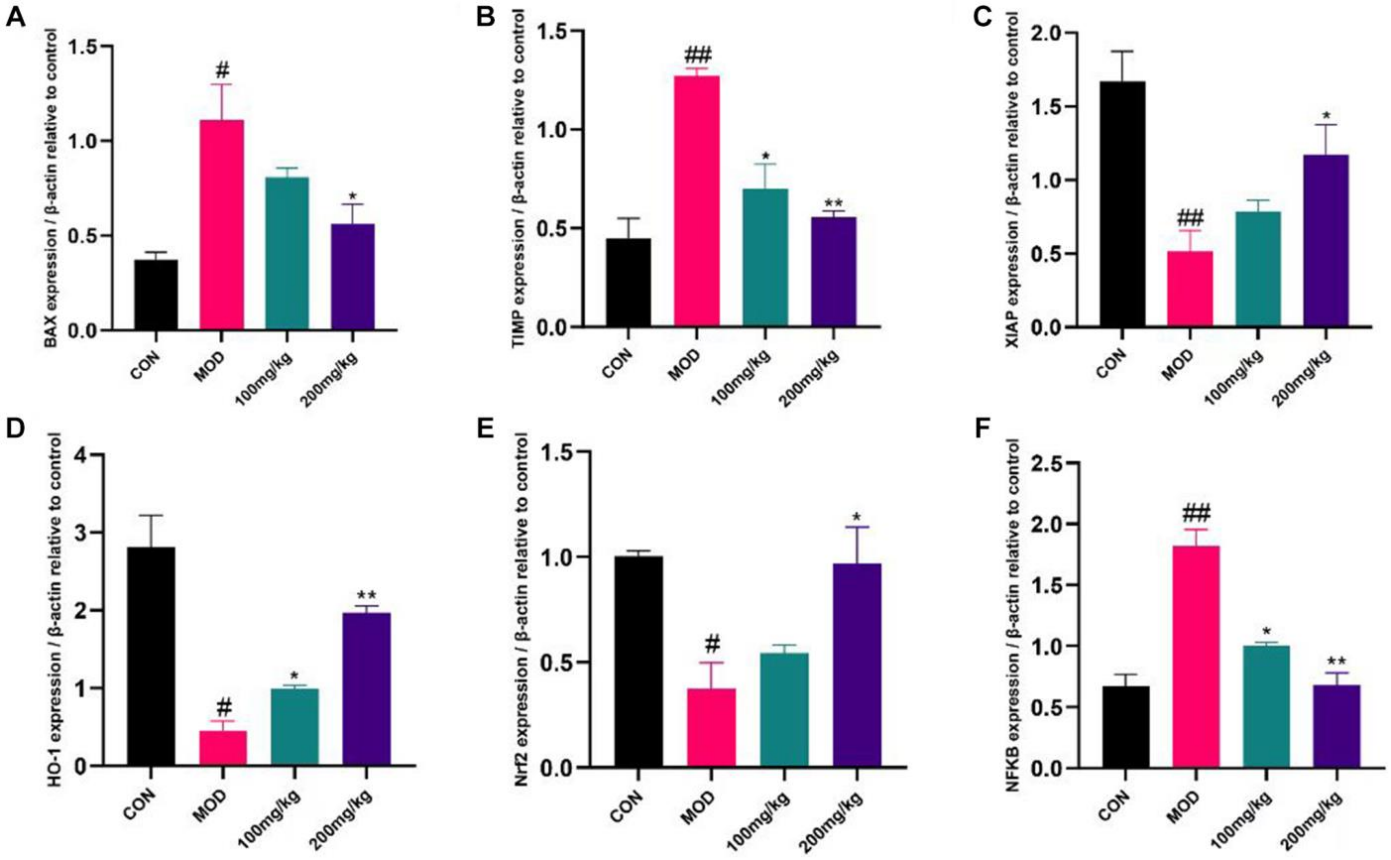
**Expression of apoptosis, inflammatory factors and anti-apoptotic markers in liver.** The liver tissue was shredded with liquid nitrogen to extract cDNA and transcribed into mRNA. The expression levels of BAX, TIMP, XIAP, HO-1, Nrf2 and NF-κB were detected by QRT-PCR. (**A**) The mRNA level of BAX. (**B**) The mRNA level of TIMP. (**C**) The mRNA level of XIAP. (**D**) The mRNA level of HO-1. (**E**) The mRNA level of Nrf2. (**F**) The mRNA level of NF-κB. ^#^*p* < 0.05, ^##^*p* < 0.01 vs. con group; ^*^p < 0.05, ^**^p < 0.01 vs. mod group.

GSLS can affect apoptosis, liver fibrosis and anti-apoptotic markers. In CPF group, apoptotic gene Bax and liver fibrosis gene TIMP were increased, while anti-apoptotic gene XIAP was decreased (Figure 8A and 8B). On the contrary, the expression of Bax and TIMP was down-regulated and the expression of XIAP was up-regulated in 100 mg/kg GSLS group. However, the expression of Bax and TIMP was down-regulated and XIAP was up-regulated in 200 mg/kg GSLS group. Compared with the control group and the GSLS pre-protected group (CPF+GSLS 100 group and CPF+GSLS 200 group), the mRNA expression of Nrf-2 and HO-1 in the CPF group was down-regulated (Figure 8C and 8D), and the mRNA expression of NFkB was up-regulated (Figure 8E). Oxidative stress was shown to occur in CPF group. In the GSLS preprotected group, liver antioxidant activity appeared to recover and NFkB expression was upregulated to normal levels (Figure 8F).

#### GSLS reduced liver necrosis in CPF-induced liver injury

**Figure 9:**
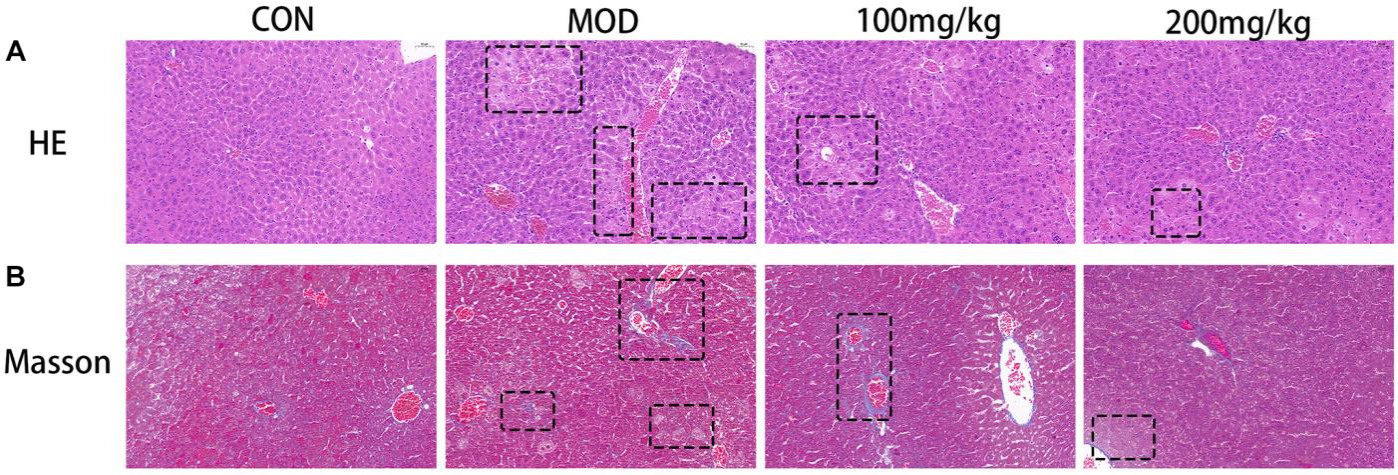
(**A**) Histological micrograph of sof hematoxylin-eosin staining liver sections. (**B**) Histological micrograph of a liver section stained by Masson.

To evaluate the role of GSLS in liver histological changes, liver sections of experimental mice were stained with trichromatic HE (Figure 9A) and Masson(B) for histological examination. Figure 9A displays the result of HE staining. The results showed that the histological structure of the liver in the control group was normal. The sections revealed well preserved cell morphology and prominent nucleus, normal liver lobule, central vein, hepatic cord, hepatic sinuses and portal vein. However, in the CPF group, the liver tissue was significantly degraded, containing vasculization, inflammation, and coagulation necrosis, and the liver was severely damaged. In the GSLS pre-protected group, fewer inflammatory cells were observed, the liver injury was reduced, and the tissue structure was normal. Steatosis and degeneration were significantly reduced in the GSLS 100 mg/kg group, and the hepatic sinus contracted slightly. The structural changes improved most significantly in the 200 mg/kg group that was brought back to normal size, indicating that GSLS preprotection alleviated CPF-induced liver injury.

As shown in Figure 9B, Masson’s trichromatic staining of liver slices showed collagen deposition (blue plaques) around portal vein and liver lobule, which was clearly CPF-induced liver fibrosis in the model group. This improved in the 100 mg/kg versus 200 mg/kg GSLS treatment group in a dose-dependent manner.

## DISCUSSION

In this study, CPF exposure can significantly increase serum ALT, AST and γGT in mice, causing serious damage to liver tissue. However, the apoptosis and oxidative stress induced by ginseng stem and leaf saponins can be antagonized by pre-administration. The histopathological sections could show the damage induced by CPF. Masson staining results demonstrated that ginseng stem saponins effectively alleviated the liver fibrosis caused by CPF.

CPF mainly causes oxidative stress injury and production of lipid peroxides, reducing antioxidant activity and generating apoptosis [34]. CPF accumulates in cells, resulting in the accumulation of intracellular ROS, which is also the main factor inducing apoptosis [35]. Flow cytometry was applied to detect the content of intracellular ROS in L02 cells, and it was found that GSLS significantly reduced the accumulation of intracellular ROS and delayed the apoptosis process. As the main site of intracellular reactive oxygen species, mitochondria exerts a key role in metabolism and energy regulation [36].

GSLS may exhibit a possible liver protective mechanism, at least partly due to certain scavenging effect of GSLS on DPPH free radical and hydroxyl free radical activities, since toxic effect of CPF on liver is mainly due to the production of free radicals, also confirming that GSLS has high antioxidant activity.

The loss of mitochondrial function is usually accompanied by a decrease in the membrane potential Δψm [37]. We employed JC-1 probe to determine mitochondrial membrane potential, and the results showed that GSLS preprotection mitigated CPF-induced mitochondrial membrane potential loss.

Oxidative stress damage occurs mainly through the production of reactive oxygen species, and may damage lipids, proteins and DNA to varying degrees [38]. Oxidative stress can induce apoptosis in a variety of ways and a number of studies have demonstrated that liver damage caused by exogenous insecticides is mainly due to oxidative stress [39–41]. This was further verified by the significant decrease of SOD and CAT levels as well as the significant increase of MDA levels in the liver tissues of mice. The antioxidant response includes a number of downstream genes that regulate oxidative stress and are regulated by Nrf2 [42]. Nrf2 also regulates heme oxygenase 1 (HO-1; HMOX1) [43]. The results of our study proved that the expression of Nrf2 and HO-1 in liver of mice preprotected by GSLS was significantly increased, indicating that Nrf2 and HO-1 played a role in regulating liver oxidative stress, which was consistent with our research results. These results demonstrate that CPF exposure can inhibit the Nrf2/HO-1 pathway in liver, and GSLS can protect Nrf2/HO-1 pathway signal transduction.

Nuclear factor kB (NF-κB) is a nuclear transcription family associated with various inflammatory responses. Studies have shown that NF-κB plays an important role in oxidative stress and regulates the expression of a series of inflammatory genes [44]. Excessive accumulation of ROS *in vivo* up-regulated NF-κB gene expression [3]. It could be found that the prior GSLS protection reduced the inflammation by down-regulating NF-κB expression.

Metalloproteinase tissue inhibitors (TIMPS) are associated with the progression of liver fibrosis. With the progression of liver fibrosis, the content of TIMP1 in liver increases [36]. As a result, we can further determine the damage of CPF to liver fibrosis by detecting the level of TIMP in the liver. In our study, the degree of liver fibrosis was significantly alleviated in mice pretreated with GSLS, confirming to the results of Masson tissue section staining.

In addition, a variety of apoptosis signaling pathways are involved in oxidative stress [45]. The Bcl-2 family includes both anti-apoptotic and pro-apoptotic members and is a pathway that can regulate mitochondrial apoptosis. Bcl-2 is an anti-apoptotic mitochondrial membrane protein, whereas Bax is a pro-apoptotic protein [46, 47]. Under the condition of intracellular oxidative stress and inflammation, the ratio of Bcl-2 to Bax protein decreases, which promotes the release of cytochrome C apoptosis factor into the cytoplasmic matrix and stimulates the activation of Caspase 3, thereby triggering mitochondrial apoptosis. CPF exposure resulted in BAX accumulation in mouse liver and induced apoptosis. XIAP acts as a chain apoptosis inhibitor and its expression level decreased. Interestingly, these changes were effectively reversed by preadministration of GSLS in mice in order to prevent oxidative stress damage and apoptosis in the liver caused by CPF exposure.

## CONCLUSION

In this study, we found that GSLS protects CPF-induced liver damage in mice by increasing antioxidant enzyme activity. And by reducing the accumulation of reactive oxygen species in cells, restoring the mitochondrial membrane potential to resist CPF damage to L02 cells.
